# Complexity Analysis of Resting-State fMRI in Adult Patients with Attention Deficit Hyperactivity Disorder: Brain Entropy

**DOI:** 10.1155/2017/3091815

**Published:** 2017-12-12

**Authors:** Gülsüm Akdeniz

**Affiliations:** Faculty of Medicine, Department of Biophysics and Yenimahalle Training and Research Hospital, Ankara Yıldırım Beyazıt University, Ankara, Turkey

## Abstract

**Objective:**

Complexity analysis of functional brain structure data represents a new multidisciplinary approach to examining complex, living structures. I aimed to construct a connectivity map of visual brain activities using resting-state functional magnetic resonance imaging (fMRI) data and to characterize the level of complexity of functional brain activity using these connectivity data.

**Methods:**

A total of 25 healthy controls and 20 patients with attention deficit hyperactivity disorder (ADHD) participated. fMRI preprocessing analysis was performed that included head motion correction, temporal filtering, and spatial smoothing process. Brain entropy (BEN) was calculated using the Shannon entropy equation.

**Results:**

My findings demonstrated that patients exhibited reduced brain complexity in visual brain areas compared to controls. The mean entropy value of the ADHD group was 0.56 ± 0.14, compared to 0.64 ± 0.11 in the control group.

**Conclusion:**

My study adds an important novel result to the growing literature pertaining to abnormal visual processing in ADHD that my ADHD patients had lower BEN values, indicating more-regular functional brain structure and abnormal visual information processing.

## 1. Introduction

Functional magnetic resonance imaging (fMRI) is among the most powerful tools for noninvasive assessment of behavior, cognition, and psychiatric disorders [1] and is used to obtain volumetric images, including time-resolution data pertaining to human brain function. In particular, the resting-state [2] fMRI technique is a preferred, alternative tool to assess brain function abnormalities in psychiatric disorders. Blood-oxygen-level-dependent signals of resting-state fMRI allow for the analysis of functional connectivity patterns within brain networks [3] and the temporal dynamics of activity fluctuations therein [4].

The most complex living structure known to man is the human brain. The reason that complexity is assessed using resting-state fMRI, with respect to psychiatric diseases, is that complex output patterns in a living system can indicate its health and robustness [5]. Complex living systems, such as the human brain, develop to possess maximum adaptive capacity [6]. The deterioration of, and reductions in, the essential functions of these complex systems, in accordance with aging and disease, is associated with a loss of complexity in the dynamics of complex physiological systems [7]. Chaotic and complex behaviors are indicative of a healthy system, whereas more predictable and regular behaviors can denote pathological states [8].

Attention deficit hyperactivity disorder (ADHD) is a common neurodevelopmental disorder that typically begins in childhood, often persists into adulthood, and is associated with consistent deficits in error processing and inhibition and regionally decreased grey matter volume [9, 10]. Although diagnosis is made on a behavioral basis, cognitive deficits may also be significant, especially in terms of executive function [11, 12] and attentional processes [13, 14]. Numerous neuroimaging studies have been conducted on ADHD; Bush [5, 15] reviewed several functional imaging studies and observed a consistent pattern of frontal dysfunction in ADHD patients. However, few studies have examined both frontal and other brain regions [16].

Entropy, a powerful indicator of irregularity in a system [17], is not associated with the value of a random variable, but depends only on the distribution of values. Entropy can characterize the level of chaos in, and complexity of, a system, within the context of information theory [18]. In medical image processing applications, entropy provides a measure of the heterogeneity of the distribution of data in the image matrix. When all data are identical, the entropy value is zero; this value increases commensurately with differences in the data and its distribution. Entropy is a useful tool in neuroscience research for obtaining meaningful results from the analysis of fMRI data [19–21]. In previous fMRI studies, entropy was considered first as an innovative, alterative indicator [22] and then as a means of detecting activation [1]; it is viewed currently as a potential marker of brain diseases [23, 24]. Measurements of brain entropy (BEN) may be used to make inferences regarding brain status and alterations due to disease [21].

The aim of the present study was (i) to determine the complexity of visual brain activity by calculating brain entropy and (ii) to assess differences between attention deficit hyperactivity disorder patients and healthy controls in terms of brain status.

## 2. Material and Methods

### 2.1. Resting-State fMRI Data Acquisition

The fMRI images used in this study were downloaded freely from the website of the 1000 Functional Connectomes Project [25]. The gender distribution in the ADHD group (*n* = 25) was as follows: 20 males and 5 females, ranging in age between 20 and 50 years. The following scanning parameters were used: TR = 2; # slices = 39; and # timepoints = 192. The second group, comprising healthy volunteers (*n* = 20), included 8 males and 12 females between 18 and 46 years of age; in this group, the following scanning parameters were applied: TR = 2; # slices = 33; and # timepoints = 175.

All research conducted by ADHD-200 contributing sites was conducted with local IRB (institutional review board) approval and contributed in compliance with local IRB protocols. All data distributed via the International Neuroimaging Data-Sharing Initiative is fully anonymized in compliance with HIPAA (The Health Insurance Portability and Accountability Act) Privacy Rules.

### 2.2. Image Analysis

FEAT fMRI preprocessing analysis of the downloaded 4D fMRI data sets was performed using the FSL [26] software package. Standard preprocessing analysis includes head motion correction, temporal filtering, and spatial smoothing process. All data were filtered according to a high-pass filter cut-off value of 100 s, motion-corrected, and smoothed with a Gaussian kernel using a full width at half maximum value of 5 mm. Independent component analysis (ICA) is a computational technique for revealing hidden factors that underlie fMRI raw data. In this study, the MELODIC (Multivariate Exploratory Linear Optimized Decomposition into Independent Components) independent component analysis (ICA) technique was used to separate single fMRI data sets into different spatial and temporal components in analyzing the fMRI data. Each subject's structural image was registered to standard Montreal Neurological Institute space (i.e., to the MNI152 template) for the purposes of spatial normalization. As a result of the analysis, components 17–44 were produced for each patient.

### 2.3. Entropy Calculation

Various components for each participant, including one pertaining to the most meaningful pattern of visual activation, were selected to assess complexity (one participant was excluded from the study, because no meaningful visual activation could was detected). The brain entropy values of the two sequential images most closely matched and, with the minimum degree of noise, were calculated for each subject using a program written using the MATLAB GUI (Mathworks, Inc.) software package [27]. This program was used to evaluate the brain entropy mapping from fMRI data. Entropy was calculated using Shannon's entropy as follows:

(1) $$\begin{array}{c} \text{BEN}=-\overset{256}{\underset{i=1}{\sum }}p\left(\;i\;\right)\log_{2}\;p\left(\;i\;\right), \end{array}$$

where *p*(*i*) is the normalized probability function of the pixel intensity *i* of the image.

Shannon entropy is a measure of how much information is required, on average in a given discrete probability distribution. The probability function of the pixel intensities was acquired and normalized by dividing by the total pixel number.

**Figure 1:**
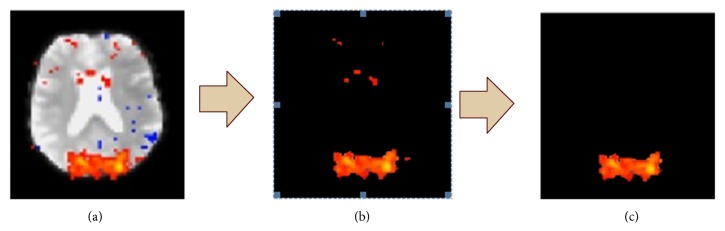
Flowchart of the color image segmentation process used to extract visual activation data: (a) fMRI image, (b) color segmentation process, and (c) a visual activation data.

Directly calculating BEN from fMRI data is challenging and prone to inaccuracy due to the effects of the image pixels that represent the brain tissue and to their negative correlation with entropy values. To overcome this, I segmented the images selected for BEN analysis according to their color. Profiles of five important colors were given in color image segmentation process: the background color, white, grey, blue, and red (Figure 1(a)). A display of these segmentation techniques and their application to extract white, grey, and blue colors is shown in Figure 1(b). After extraction, the remaining red color signals which do not represent the activation of occipital lobe had to be considered as an artifact and removed from the images in Figure 1(c). Figure 1 provides a flowchart of the color image processing steps necessary to calculate BEN.

### 2.4. Statistical Analysis

Parametric statistical analysis was performed using the Statistical Package for Social Sciences (SPSS16.0; Chicago, IL, USA) software. *t*-test was conducted for differences between the mean entropy values in the ADHD group and control group. Mann–Whitney *U* test was performed to reveal the group differences between the patient and control groups in gender and age. The relationship between the BEN and group and between gender and group was determined by using Pearson's chi-squared test.

## 3. Results

**Figure 2:**
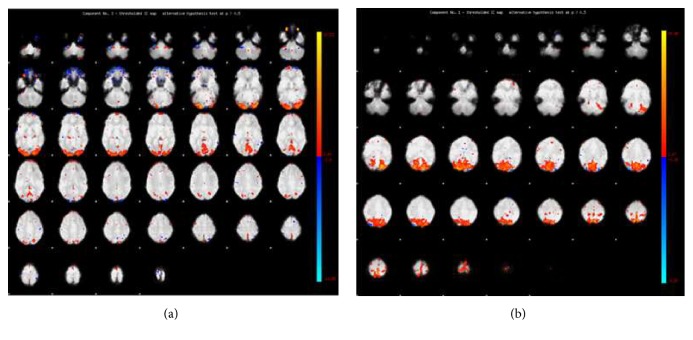
Images produced by fMRI analysis denoting visual activation in (a) an ADHD patient and (b) a control participant.

I have achieved fMRI components and Figure 2 depicts two example fMRI components. It should be noted that I selected and used, during calculation of BEN, only components exhibiting meaningful visual activation, such as those shown below.

**Figure 3:**
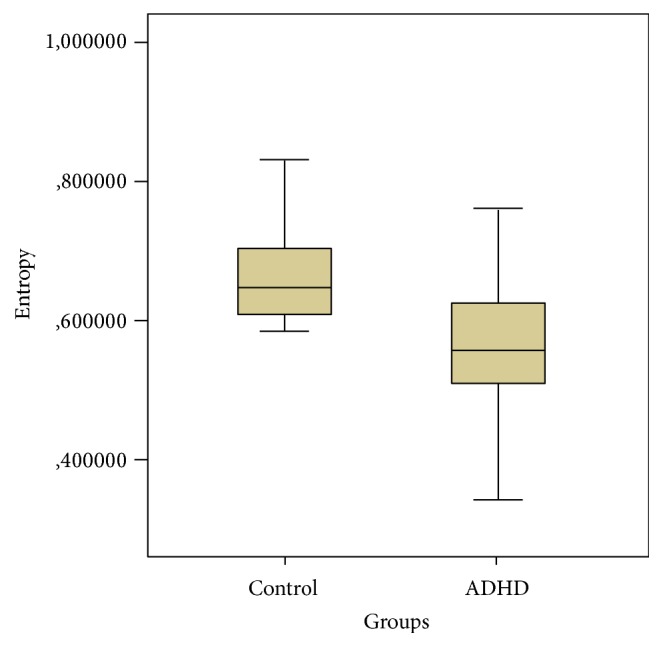
BEN bars of the ADHD patient and control groups.

The BEN bars of the ADHD patients and controls are depicted in Figure 3. The mean entropy value of the ADHD group was 0.56 ± 0.14, compared to 0.64 ± 0.11 in the control group; this difference was significant (*p* = 0.008).

Table 1 lists the comparisons results between the patient and control groups. There were significant group differences in gender (*p* = 0.010) and age (*p* = 0.045). Data are provided as means ± SD.

On correlation analysis, there was a negative relationship between BEN and group (*r* = −0.306; *p* = 0.043) and between gender and group (*r* = −0.390; *p* = 0.009).

## 4. Discussion

Throughout the past decade, the majority of studies on psychiatric diseases have been fMRI-based investigations [10, 28, 29]. I studied ADHD using this technique due to the importance of functional connectivity in psychiatric diseases and because of the ability of fMRI to index this connectivity and activity within the brain. I employed BEN mapping, of functional brain connectivity, to better understand the disease.

Complexity analysis of functional brain structure [30] is a promising tool with which to examine functional brain connectivity at an organizational level [31]. The degree of complexity is associated with the number of brain connections; decreased connectivity indicates lower complexity, and increased connectivity reflects greater complexity. Numerous studies on brain activity in ADHD have been performed using functional neuroimaging techniques other than fMRI, such as electroencephalography and magnetoencephalography [32, 33]. Gómez et al. [32] demonstrated that MEG recordings of ADHD patients were more-regular compared to recordings obtained in a control group; furthermore, there were significant differences among these groups in five brain regions, that is, anterior, central, posterior, left lateral, and right lateral areas. Sokunbi et al. [34] used resting-state fMRI to demonstrate reduced complexity in the brain activity of adult ADHD patients compared to healthy, age-matched controls. van den Heuvel and Hulshoff Pol [3] suggested resting-state fMRI studies examining functional connectivity have provided a new and promising platform to examine hypothesized disconnectivity effects in psychiatric brain diseases. There has been a reawakening of interest in an alternative approach that focuses on the resting state [15]. I investigated functional brain connectivity in occipital areas using resting-state fMRI; in contrast to previous studies, I observed a more-regular pattern of complexity, in the context of visual activities, in the patient group compared to the control group. A previous structural study reported decreased total brain and occipital lobe volume in ADHD patients [35, 36]. I suggested that this reduction in occipital lobe volume may account for the reduced functional brain connectivity, and more-regular pattern of complexity, observed in ADHD.

BEN can quantify the complexity of the functional architecture of the human brain [30], such that the BEN values I obtained during my complexity analysis provide a physiologically and functionally meaningful account of the brain activity of both ADHD patients and controls. My BEN results, obtained from fMRI image analyses, also show that individuals with ADHD exhibit lower entropy in particular brain regions compared to controls, thereby indicating a more-regular pattern of complexity. This is consistent with Goldberger and Lipsitz's model of robustness [5, 7, 37–39] in which the complexity of a system's physiological output decreases commensurately with greater age and disease. Therefore, I proposed that entropy may represent a useful indicator for research on various brain states.

Several researchers have reported abnormal frontal-striatal brain function in patients with ADHD [40]. However, a growing number of studies also indicate abnormal posterior brain function, and associated abnormalities, in early-stage sensory information processing [41]. According to a recent meta-analysis of fMRI studies examining task-based cognition in ADHD, functional abnormalities in the visual cortex may represent a key finding in ADHD [41]. My study adds an important novel result to the growing literature pertaining to abnormal visual processing in ADHD; that is, my ADHD patients had lower BEN values, indicating more-regular functional brain structure and abnormal visual information processing. Furthermore, I demonstrated abnormalities in brain function in the occipital region using resting-state fMRI. Abnormal visual information processing in ADHD has been also identified by previous studies, consistent with my results [42, 43].

Numerous studies indicated that ADHD patients exhibit gender differences in clinical and sociodemographic characteristics [44–46]. Taken together, the results of these studies are in general agreement in terms of suggesting that young female ADHD patients exhibit lower ratings on hyperactivity, inattention, impulsivity, and externalizing problems, in addition to greater intellectual impairments and internalizing of problems, compared to young males with ADHD [37]. The prevalence of ADHD in the adult population is 4.4% in the US, of whom 38% are female and 62% are male (National Resource Center on ADHD). These data are generally supported by my study, with respect to gender differences between the study groups, but not in terms of BEN values. These results support Rubin and colleagues' study; they suggested that fMRI applications of complexity have dealt with both between the voxel and between-subject differences [30].

My BEN results indicate that excessive orderliness is not advantageous and in fact indicates abnormal function; greater complexity indicates a healthier system. I believe that using entropy analyses during fMRI may be of benefit to research in various pathologic and nonpathologic areas. Furthermore, I suggest that BEN can index brain activity and may help to determine abnormalities in brain activity, for example, in pilot studies of drug addiction [47].

In conclusion, the present study successfully demonstrated a reduction in BEN in ADHD patients compared to healthy controls, by calculating signal entropy (Shannon entropy) in accordance with the level of complexity of resting brain activity. This result supports the notion that the complexity of resting brain activity can be used as an indicator of ADHD.

## Figures and Tables

**Table 1:** Comparison of group characteristics.

	ADHD (*n* = 25)	Control (*n* = 20)	*Z*/*χ*^2^	*p*
Age	34.52 ± 9.54	29.32 ± 10.02	−2.007	0.045
Gender^*∗*^ (male/female)	20/5	8/11	6.699	0.010
Brain entropy (BEN)	0.56 ± 0.14	0.64 ± 0.11	−2.666	0.008
ADHD-RS				
Inattentive	16.79 ± 4.90	-	-	-
Hyperactive/impulsive	13.13 ± 5.17	-	-	-
Total	29.92 ± 8.75	-	-	-
ACDS				
Inattentive	6.59 ± 2.52	-	-	-
Hyperactive/impulsive	7.82 ± 1.33	-	-	-

Mann–Whitney *U* test, ^*∗*^Pearson's chi-squared test; ADHD-RS: ADHD-Rating Scale; ACDS: Adult ADHD Clinical Diagnostic Scale.
